# Myocardial infarction care in low and high socioeconomic environments: claims data analysis

**DOI:** 10.1007/s12471-023-01813-z

**Published:** 2023-10-12

**Authors:** Alexander D. Hilt, Victor A. W. M. Umans, Tessel N. E. Vossenberg, Martin J. Schalij, Saskia L. M. A. Beeres

**Affiliations:** 1 Department of Cardiology, Leiden University Medical Centre, Leiden, The Netherlands; 2 Department of Cardiology, Noordwest Ziekenhuisgroep, location Alkmaar, Alkmaar, The Netherlands; 3 Department of Cardiology, Medisch Centrum Leeuwarden, Leeuwarden, The Netherlands

**Keywords:** Claims data, Myocardial infarction, Socioeconomic status, Revascularisation, Medication

## Abstract

**Background:**

To date, claims data have not been used to study outcome differences between low and high socioeconomic status (SES) patients surviving ST-elevation myocardial infarction (STEMI) and non-ST-elevation myocardial infarction (NSTEMI) in the Netherlands.

**Aim:**

To evaluate STEMI and NSTEMI care among patients with low and high SES in the referral area of three Dutch percutaneous coronary intervention (PCI) centres, using claims data as a source.

**Methods:**

STEMI and NSTEMI patients treated in 2015–2017 were included. Patients’ SES scores were collected based on their postal code via an open access government database. In patients with low (SES1) and high (SES4) status, revascularisation strategies and secondary prevention medication were compared.

**Results:**

A total of 2065 SES1 patients (age 68 ± 13 years, 58% NSTEMI) and 1639 SES4 patients (age 68 ± 13 years, 63% NSTEMI) were included. PCI use was lower in SES1 compared to SES4 in both STEMI (80% vs 84%, *p* < 0.012) and NSTEMI (42% vs 48%, *p* < 0.002) patients. Coronary artery bypass grafting was performed more often in SES1 than in SES4 in both STEMI (7% vs 4%, *p* = NS) and NSTEMI (11% vs 7%, *p* < 0.001) patients. Optimal medical therapy use in STEMI patients was higher in SES1 compared to SES4 (52% vs 46%, *p* = 0.01) but comparable among NSTEMI patients (39% vs 40%, *p* = NS). One-year mortality was comparable in SES1 and SES4 patients following STEMI (14% vs 16%, *p* = NS) and NSTEMI (10% vs 11%, *p* = NS).

**Conclusion:**

Combined analysis of claims data and area-specific socioeconomic statistics can provide unique insight into how to improve myocardial infarction care for low and high SES patients.

**Supplementary Information:**

The online version of this article (10.1007/s12471-023-01813-z) contains supplementary material, which is available to authorized users.

## What’s new?

- Low socioeconomic status is linked to worse outcomes after ST-elevation myocardial infarction (STEMI) and non-ST-elevation myocardial infarction (NSTEMI).
- Insight into differences in the treatment of STEMI and NSTEMI patients at a socioeconomic and regional level is crucial to improve survival in these patients, but not easily achieved.
- Healthcare claims data coupled to open-access governmental socioeconomic data at postal-code level can be used to assess STEMI and NSTEMI healthcare in the Netherlands in a novel and accessible way.
- Regional Dutch STEMI and NSTEMI patient data show that coronary artery bypass grafting is performed more frequently in low socioeconomic classes and secondary preventive medication use is modest among higher socioeconomic classes.
- The outcome of this proof-of-concept study provides direction for improving regional myocardial infarction care at a postal-code level, for both low and high socioeconomic classes.

## Introduction

Socioeconomic status (SES) provides insight into the welfare level of an area and its inhabitants regarding various components, including education, income and employment [1, 2].

In patients with coronary artery disease, low SES is associated with increased morbidity and mortality, and is therefore a risk factor for a worse outcome after ST-elevation myocardial infarction (STEMI) and non-ST-elevation myocardial infarction (NSTEMI) [3, 4].

Improving the socioeconomic environment requires a conjoined effort by civilians, healthcare professionals and the government. Furthermore, cardiologists and allied professionals should be concerned with the evaluation and improvement of clinical care after myocardial infarction in different socioeconomic environments. Prospective registry-based studies can provide insight into demographic, ethnic and SES differences among myocardial infarction patients [5]. Contemporary databases, such as claims data registries, provide a unique alternative to assess outcomes of patients surviving myocardial infarction, as these databases provide accurate ‘real-world’ data [6–10]. To date, claims data have not been used to study differences between low and high SES myocardial infarction patients in the Netherlands.

The current ‘proof of concept’ study, combining claims data with socioeconomic data, evaluates STEMI and NSTEMI care and outcome among patients with the lowest and highest SES in the referral area of three Dutch percutaneous coronary intervention (PCI) centres. In particular, revascularisation strategies, secondary preventive medication use and 1‑year mortality after STEMI and NSTEMI are analysed. The results of the current study may ultimately help to develop improvement strategies for modifiable factors on a regional level (infographic).

## Methods

In the Netherlands, hospital and pharmaceutical claims data are sent to patients’ insurance companies and subsequently collected in the central database of the insurance companies. The current study was performed in close collaboration with the Dutch National Healthcare Institute (ZINL), which advises the Dutch government and has access to both hospital and pharmaceutical claims databases.

### Study design

This is a retrospective cohort study. Pseudo-anonymous and encrypted patient data were used. Dutch law states that ethical review and approval is not necessary for this type of analysis. Access to the claims data of each hospital was granted after obtaining signed consent from the head of the cardiology department of each participating hospital.

### Study population and data collection

**Fig. 1:**
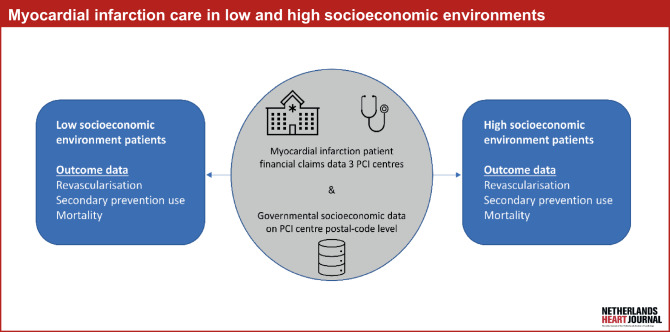
Infographic: Study overview. Assessing modifiable factors in locoregional myocardial infarction care through claims data combined with governmental socioeconomic statistics. *CABG* coronary artery bypass grafting, *PCI* percutaneous coronary intervention

All adult myocardial infarction patients treated in three participating Dutch PCI centres (two on-site: Leeuwarden Medical Centre and Leiden University Medical Centre; one off-site: PCI centre, Northwest Hospital Group, Alkmaar) were included. Patient inclusion criteria were: (1) diagnosis of STEMI (claims code 0320.11.204) or NSTEMI (claims code 0320.11.205) between 1 January 2015 and 31 December 2017, (2) follow-up of at least 1 year in one of three participating hospitals and (3) patients residing in postal-code areas surrounding the participating hospitals (Appendix 1, Electronic Supplementary Material). Postal codes were used to assign patients to SES classes, SES1 being the lowest and SES4 the highest class. The aim of the current study was predominantly to compare myocardial infarction care between SES1 and SES4. Demographic data, revascularisation strategies, medication use and 1‑year mortality were evaluated in all patients. Mortality at different intervals (i.e. after 1 week or 2 months) was not included. Patients who had more than one myocardial infarction during the study period were excluded. Figure 1 gives an overview of the aforementioned study approach.

### SES score

SES scores for all postal codes in the Netherlands are calculated by the Dutch government and are available online. SES scores are based on yearly income, employment status and education [11, 12]. The average national SES score has a given numerical value of 0. This vector changes according to diverse variables: low income and low educational level result in a low SES score, higher income and educational level result in a higher SES score. Between 2015 and 2017, Dutch SES scores were divided into four quartiles, with the lowest being SES1 and the highest SES4. The quartiles were classified as SES1: −8.19 to −0.57; SES2: −0.57 to 0.15; SES3: 0.15–0.71; SES4: 0.71–2.93. Patients of intermediate SES classes (SES2, SES3) were not analysed, as this was not the aim of the study.

### Outcome measure—revascularisation

Revascularisation through PCI or coronary artery bypass grafting (CABG) was assessed. PCI was defined as patients ‘having undergone angiography with placement of a stent or balloon angioplasty within 4 days of the initial STEMI or NSTEMI diagnosis registration’. The cut-off of 4 days was based on European and American guidelines, which recommend an invasive strategy within the first 72 h (the acute or semi-acute phase of the STEMI or NSTEMI) [13, 14]. CABG was defined as patients ‘having had bypass surgery within 30 days of initial STEMI or NSTEMI diagnosis registration’. PCI or CABG at different time intervals were not analysed in the current study.

In addition, the total number of repeat PCI or repeat CABG procedures was not analysed.

### Outcome measure—optimal medical treatment

Optimal medical therapy (OMT) after myocardial infarction was defined as the combined use of aspirin, a P2Y12 inhibitor, a statin, a beta blocker and an angiotensin-converting enzyme/angiotensin II inhibitor as recommended by international STEMI and NSTEMI guidelines [13, 14]. OMT use was measured 1 year after the initial STEMI or NSTEMI diagnosis registration, extracted from the national pharmacy database as a combination of anatomical therapeutic chemical (ATC) codes together with the daily defined dosage (DDD) of that type of medication. The DDD for each medication is determined by the World Health Organisation Collaborating Centre for Drug Statistics Methodology. It is the assumed average maintenance dose for a drug used for its main indication in adults. For each drug, a DDD threshold of at least 10 dosage units during 1 year was used, i.e. a patient had to take at least 10 units (for example metoprolol 50 mg once daily) over a period of 1 year to be deemed a ‘metoprolol user’. The use of these ATC and DDD definitions per medication has been validated in previous studies [6–9].

### Statistical analysis

Data are presented as absolute numbers and as a proportion of the total population (%). Proportion comparisons were done by a χ^2^ test. A *p*-value of 0.05 was considered statistically significant. Data were analysed using IBM SPSS Statistics, version 25 (IBM Corp., Armonk, NY, USA).

## Results

### Study population

During the study period, 7264 unique myocardial infarction patients were treated in the participating hospitals. Of these, 2065 (28%) were SES1 patients and 1639 (23%) were SES4 patients (Tab. 1). The majority were NSTEMI patients: 1195 (58%) in the SES1 group and 1040 (63%) in the SES4 group. Mean age at the time of STEMI and NSTEMI was similar in SES1 and SES4 patients (Tab. 1). There were significantly fewer males in the SES1 group than in the SES4 group of STEMI patients (64% vs 74%, *p* < 0.001), but the difference was smaller among NSTEMI patients (65% vs 66%, *p* = 0.02). One-year mortality in SES1 and SES4 was similar in STEMI (14% vs 16%) and NSTEMI (11% vs 10%) patients (*p* = NS).

**Table 1:** Demographics of acute myocardial infarction (*AMI*) patients within socioeconomic classes in 2015–2017

SES	SES1	SES4	*p*-value
AMI (*n*)	2065	1639	
STEMI (*n*, %)	870 (42)	599 (37)	
Age (mean, SD)	66.5 (13)	66.7 (13)	NS
Male (*n*, %)	551 (64)	322 (74)	< 0.001
1‑year mortality (*n*, %)	121 (14)	70 (16)	NS
NSTEMI (*n*, %)	1195 (58)	1040 (63)	
Age (mean, SD)	70.5 (13)	70.4 (12)	NS
Male (*n*, %)	759 (65)	612 (66)	0.02
1‑year mortality (*n*, %)	134 (11)	94 (10)	NS

*SES* socioeconomic status, *STEMI* ST-elevation myocardial infarction, *NSTEMI* non-ST-elevation myocardial infarction

### Revascularisation by SES

**Fig. 2:**
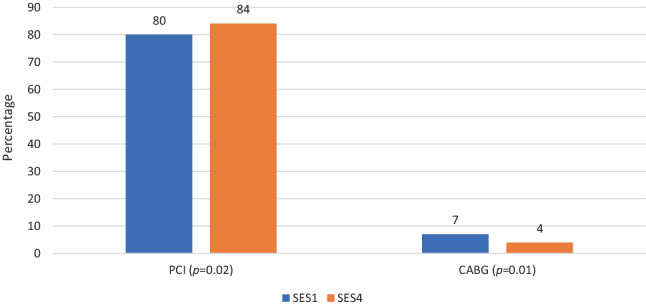
Total number of percutaneous coronary interventions (*PCI*) and coronary artery bypass grafts (*CABG*) performed in ST-elevation myocardial infarction (*STEMI*) patients stratified by socioeconomic status (*SES*)

**Fig. 3:**
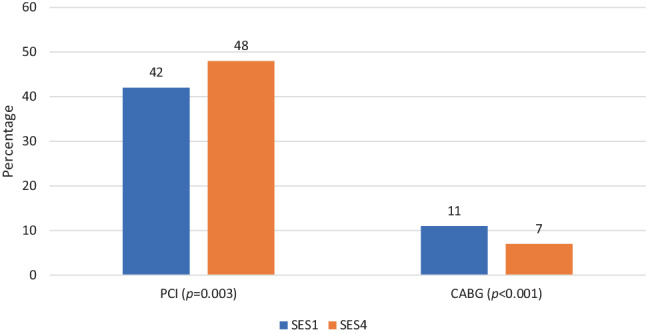
Total number of percutaneous coronary interventions (*PCI*) and coronary artery bypass grafts (*CABG*) performed in non-ST-elevation myocardial infarction (*NSTEMI*) patients stratified by socioeconomic status (*SES*)

Figures 2 and 3 show treatment by PCI and CABG among STEMI and NSTEMI patients stratified by SES. In both STEMI and NSTEMI patients, PCI procedures were performed significantly less frequently in SES1 patients than in SES4 patients (*p* = 0.02 for STEMI and *p* = 0.003 for NSTEMI). In contrast, in both STEMI and NSTEMI patients, CABG procedures were more often performed in SES1 patients than in SES4 patients (STEMI, *p* = 0.01; NSTEMI, *p* < 0.001).

### Medication usage by SES

**Fig. 4:**
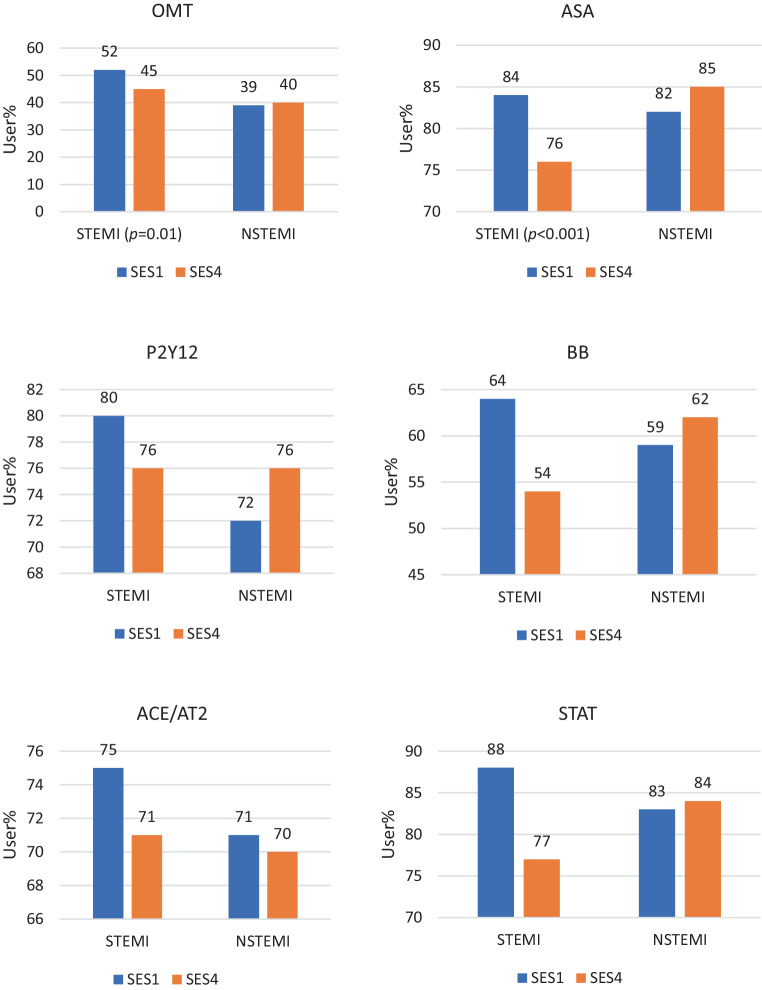
Optimal medical therapy (*OMT*) and individual medication use among ST-elevation myocardial infarction (*STEMI*) and non-ST-elevation myocardial infarction (*NSTEMI*) patients stratified by socioeconomic status (*SES*). *ACE/AT2* angiotensin converting enzyme-inhibitor or angiotensin II antagonist, *ASA* aspirin species, *BB* beta blocker, *P2Y12* P2Y12 inhibitor, *STAT* statin

Figure 4 shows medication use by STEMI and NSTEMI patients stratified by SES. Following STEMI, SES1 patients more frequently used complete OMT as compared to SES4 patients (52% vs 46%, *p* = 0.01).

The higher rate of medication use by SES1 patients was driven by a higher use of aspirin (84% vs 76%, *p* < 0.001), P2Y12 inhibitors (80% vs 76%, *p* = 0.03), beta blockers (64% vs 54%, *p* < 0.001) and statins (84% vs 77%, *p* < 0.001).

Following NSTEMI, 39% of SES1 patients used complete OMT as compared to 40% of SES4 patients (*p* = NS). The only difference in NSTEMI patients concerned P2Y12 inhibitors, with lower use in SES1 patients (72% in SES1 vs 76% in SES4, *p* = 0.01).

## Discussion

The main findings of the current study can be summarised as follows. First, following both STEMI and NSTEMI, SES1 patients were less frequently treated by PCI and more frequently by CABG as compared to SES4 patients. Second, the use of OMT is moderate in both SES1 and SES4 patients. Following STEMI, SES1 patients more frequently used complete OMT as compared to SES4 patients. Following NSTEMI, there was no difference in the rate of complete OMT use. Lastly, combining claims data with area-specific socioeconomic statistics is an efficient method to analyse cardiac care on a locoregional level in a unique way.

Our study shows that low SES STEMI and NSTEMI patients are treated by CABG more frequently than high SES patients. High CABG frequencies among low SES patients could be the result of more complex coronary lesions in this group. Previous studies have shown that, overall, low SES patients exhibit more risk factors (smoking, hypertension, hypercholesterolaemia and diabetes) at presentation, resulting in multivessel disease rather than one-vessel disease [15–17]. The relatively high frequency of CABG procedures in the low SES patients hints at the possibility of this population being unhealthier. Importantly, the findings of the current study provide a rationale for a conjoined initiative by cardiologists, family physicians and healthcare insurance companies to improve lifestyle in low SES regions.

OMT use was modest in STEMI and NSTEMI patients, among both low and high SES classes. This finding is not in line with a more recent study by Lee et al., which addressed the use of antihypertensive medication in event-free patients from different socioeconomic backgrounds, showing that low SES patients were less reluctant to adhere to medication [18]. However, an important difference from this study is that the event-free population is not comparable to our cardiovascular-event population. Furthermore, the presented study is in line with previous Dutch [6, 8, 19], British and American studies [20–22], all stressing the need for increased awareness of medication adherence after myocardial infarction. When addressing individual medication use after STEMI, foremost aspirin, beta blockers and statins are used more frequently by low SES patients than by high SES STEMI patients. In NSTEMI patients, low and high SES patients show a comparable low-usage pattern. Although some previous studies show that low SES has a negative effect on medication adherence [18, 23, 24], others have shown a negative effect of high SES [25, 26] or no effect of SES on medication adherence [27]. The reason for the observed difference in our study is difficult to ascertain, as no clinical data such as allergies or side-effect patterns were used. Lower use of cardioprotective medication in high SES STEMI patients can be related to an unwillingness to take medication because of doubts or a fear of side-effects, as observed in more highly educated patients [21, 28]. It could, however, also be related to a healthier lifestyle and less prevalent risk factors. Equally, a higher adherence among low SES STEMI patients could result from more CABG procedures being performed, having an effect on medication adherence in younger, low SES patients. As medication adherence is indispensable for survival after myocardial infarction, initiatives focusing on medication adherence in the high SES patients in the outpatient follow-up by cardiologists and primary care physicians are warranted.

### Future perspectives

For the first time in Dutch healthcare, claims data were combined with area-specific socioeconomic statistics. These data illustrate that treatment patterns and healthcare use in specific regions and specific patient groups can be analysed by this approach. This type of research differs from previously performed ‘causality studies’ assessing the impact of low SES on mortality or adverse events after myocardial infarction [16, 28, 29].

Our findings stress the importance of primary prevention programmes for myocardial infarction patients in low SES regions and equally provide a rationale for the development of quality improvement programmes which focus on medication adherence after myocardial infarction among inhabitants of high SES regions. By aiming at inhabitants of specific regions, scarce healthcare resources are spent more effectively and care becomes more patient-centred. For example, medication use after myocardial infarction could be the topic of patient-education gatherings in general practices or cardiac outpatient clinics to increase awareness. Furthermore, understanding the patient’s experience regarding medication use has to be addressed further on a qualitative level via, for example, questionnaires and interviews, as a local study has shown [30].

### Limitations

Some limitations should be considered when interpreting the results. First, the study uses observational data. Second, the SES score used is a postal-code average and not derived on a personal level: some SES4 patients might live in an SES1 area and vice versa. Additionally, yearly income or level of education were not separately collected per patient in our study. Third, the level of clinical details per patient is limited. Accordingly, completeness of revascularisation, ventricular function or infarct size are not included and commonly used risk scores (e.g. GRACE risk score) cannot be applied to our study population. Lastly, the total number of CABG procedures performed in our population could be underestimated because one off-site PCI centre was included.

## Conclusion

Claims data combined with area-specific socioeconomic statistics provide unique insight into regional myocardial infarction healthcare in the Netherlands. A focus on primary prevention strategies and improved medication adherence in low and high SES classes, respectively, could be the next steps toward further improving regional cardiovascular healthcare.

### Supplementary Information

Appendix 1. Included zip-codes of analyzed PCI centers
