# Supplementary material for: Myocardial infarction care in low and high socioeconomic environments: claims data analysis
Source: Neth Heart J. 2023 Oct 12;32(3):118–24. doi: 10.1007/s12471-023-01813-z (PMC10884367; doi:10.1007/s12471-023-01813-z)
Supplement: Supplementary file 1 — Appendix 1. Included zip-codes of analyzed PCI centers [file 12471_2023_1813_MOESM1_ESM.docx]

**Appendix 1. Included zip-codes of analyzed PCI centers.**
**Leeuwarden Medisch Centrum (on-site PCI center)**

- 8411 – 8495
- 8601 – 8651
- 8851 – 8584
- 8701 – 8773
- 8800 – 8896
- 8911 – 8941
- 9001 - 9089
- 9100 – 9178
- 9251 - 9265
- 9257 – 9269
- 9271 – 9298
- 9851 - 9853
- 9950

**Leiden University Medical Center (on-site PCI center)**

- 2171 - 2191
- 2201 - 2254
- 2311 – 2361

**Noordwest Ziekenhuisgroep – Alkmaar (off-site PCI center)**

- 1483 – 1486
- 1536
- 1631 - 1652
- 1711 - 1797
- 1811- 1873
- 1901 – 1992
- 2071 - 2082
